# FOODWISE (Fostering Optimal Outcomes through Dietary Wisdom, Integration, Spirituality, and Emotionality): A New Model of Integrative Culinary Medicine

**DOI:** 10.1089/jicm.2023.0751

**Published:** 2024-02-14

**Authors:** Melinda Ring, Darshan H. Mehta, Iman Majd, Anna Balabanova Shannahan

**Affiliations:** ^1^ Osher Center for Integrative Health, Northwestern University Feinberg School of Medicine, Chicago, IL, USA.; ^2^ Harvard Medical School, Boston, MA, USA.; ^3^ Department of Medicine, Benson-Henry Institute for Mind Body Medicine, Massachusetts General Hospital, Boston, MA, USA.; ^4^ Osher Center for Integrative Health, Brigham & Women's Hospital and Harvard Medical School, Boston, MA, USA.; ^5^ Osher Center for Integrative Health, University of Washington, Seattle, WA, USA.


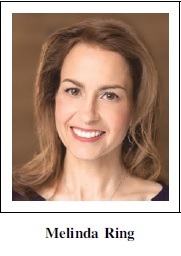



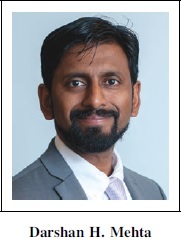



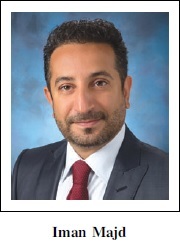



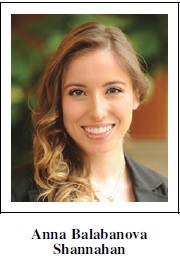


Nutrition training for health professionals has traditionally put the spotlight on the physical impacts of food, often sidelining its broader influences on our well-being.^1^ While we've come a long way in acknowledging that food is more than just fuel, many current educational approaches still focus predominantly on the physiological, neglecting the spiritual, cultural, and emotional dimensions of eating. This narrow scope calls for a paradigm shift, one that integrative culinary medicine (ICM) is uniquely positioned to provide.

Many health professionals, especially physicians, are underprepared to counsel patients about nutrition and food. This deficiency was publicly acknowledged in the past year by the passage of the Bipartisan Resolution to Bolster Nutrition Education for Medical Professionals^2^ and the Summit on Medical Education in Nutrition hosted by leading graduate and undergraduate academic medicine organizations.^3^ While awaiting further direction, academic programs across the globe have proliferated culinary medicine courses to train health professionals about real-world nutrition through the lens of cooking. These culinary medicine programs have shown a positive short-term impact on provider attitudes, knowledge, and patient health behaviors.^4,5^

As new nutrition curricula are being developed, there may be additional benefits to addressing nutrition and health through a patient-centric and holistic model. A patient-centric model prioritizes the patient's needs, preferences, and values, fostering a more personalized and empowering approach to care and well-being. Focusing on the whole person rather than isolated symptoms or generic guidelines could enhance engagement, satisfaction, and long-term health outcomes.^6,7^

This more personalized approach is especially critical today as our society is becoming increasingly diverse. For instance, by 2044, >50% of all U.S. Americans will identify as part of a minority group (any group that isn't solely non-Hispanic White).^8^ The major noncommunicable diseases (NCDs), diabetes, cardiovascular diseases, cancer, chronic respiratory diseases, and mental disorders, are responsible for 71% of all deaths globally.^9^ These are causally related to modifiable risk factors, including an unhealthy diet.^10^ Globally, across both high- and low-income countries, data show NCD rates are higher in economically disadvantaged people. Moreover, there are ongoing disparities in the prevalence of multiple chronic medical conditions based on race and ethnicity.^11^ To understand and heal these disparities, health professionals need to view diet from a broader perspective rather than only food's nutritional content.

To foster health equity by addressing modifiable risk factors, there is an essential need to enhance nutrition education for health professionals to understand how to share nutrition recommendations in the context of a patient's culture, environment, and access.^12–14^ For example, the typical recommendation of the Mediterranean Diet as a universal solution to mitigate some NCDs, risks diminishing the value of other food traditions and may exclude individuals from nonwhite backgrounds.^15^

More inclusive nutrition resources and training are emerging but are still in their infancy.^16–19^ Recognizing this gap, a novel and holistic approach to dietary health is introduced here: ICM: Fostering Optimal Outcomes through Dietary Wisdom, Integration, Spirituality, and Emotionality (FOODWISE).

ICM is a unique fusion of integrative and culinary medicine that emphasizes the treatment of the “whole person” rather than focusing on isolated symptoms or diagnosis. ICM offers a nuanced understanding of how food interacts with every aspect of our lives by employing an evidence-based salutogenic model and practical skills such as cooking. This approach extends beyond nutrition as sustenance for our physical being by offering a deeper understanding of food's multifaceted role in our well-being.

## ICM Framework

**FIG. 1.**
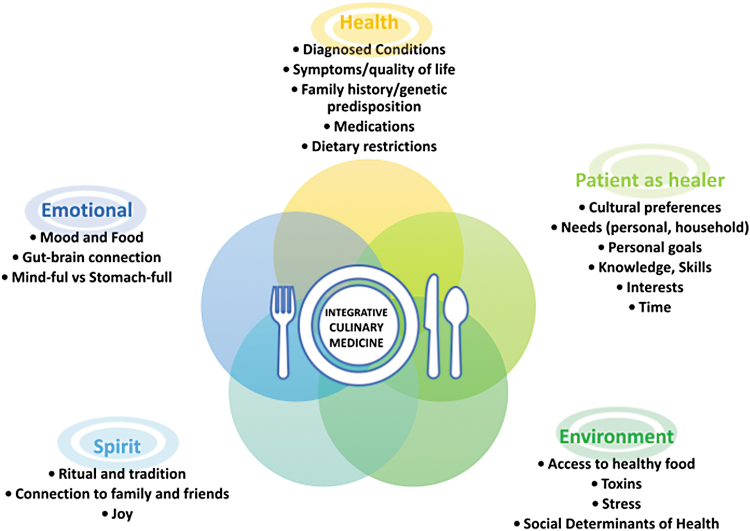
The ICM framework. This figure presents the five domains that structure the ICM approach, designed to provide a comprehensive, patient-centered, and culturally sensitive model for health care. The five domains are **Health, Patient as Healer, Environment, Spirit, Emotional.**

ICM is structured around five domains (see Figure 1):

### Health

This domain expands traditional nutritional concepts by emphasizing a broader context of health. It includes addressing diagnosed conditions, symptoms, family history, genetic predispositions, medications, and dietary modifications, focusing on disease prevention and enhancing quality of life.

### Patient as healer

This domain encourages patients to exercise autonomy and actively participate in their health. It considers cultural preferences, personal needs, goals, and abilities in fostering sustainable health-enhancing habits as related to diet.

### Environment

This domain introduces socioeconomic and sociocultural factors that impact health. It ranges from access to healthy food and exposure to toxins to societal stressors and the influences of media and advertising on dietary habits.

### Spirit

This domain recognizes the rituals and traditions around food preparation and inherent healing properties of food, emphasizing its role beyond physical nutrients. It considers food's energetic qualities, acknowledging cultural expressions that view food as therapeutic, thereby enriching the patient's relationship with eating and overall well-being.

### Emotional

The final domain explores the intricate relationships between emotional well-being and food, such as emotional eating, nutrient quality, and mood disorders, and how diet and mood are linked through the gut–brain axis. It embraces emotional factors often overlooked in traditional nutritional paradigms.

## The Transformative Potential of ICM: FOODWISE

In embracing the ICM model, we open doors to a more nuanced and compassionate approach to dietary health and wellness. The transformative potential of ICM reaches across various sectors, from education and health care to individual well-being and community health.

**Educational Institutions:** By weaving ICM into training programs, educational institutions can foster a culture of wellness and equip health professionals with the insights and skills to apply ICM principles. This approach aligns with modern health care's person-centered goals, promoting a holistic understanding of nutrition, food, and health.
**Health care Providers:** By utilizing ICM, health care professionals can gain a more comprehensive understanding of their patients, enabling them to provide holistic, personalized care that fosters patient engagement and strengthens the patient–physician relationship.
**Individuals:** ICM empowers people to make informed decisions about their dietary habits and practices, enhancing overall health and wellness, which also considers and incorporates their cultural values and beliefs system. This personalized approach can significantly benefit those with specific dietary needs or chronic illnesses.
**Community and Societal Level:** ICM has the potential to inform and shape public health policies and interventions, addressing widespread health issues related to diet, such as obesity, malnutrition, and chronic diseases.
**Culinary Professionals**: Chefs and other culinary experts can incorporate ICM principles into their practices, influencing food preparation and presentation and promoting community health and wellness.

By recognizing and integrating the multifaceted role of food, ICM offers a pathway to personalized, culturally adaptive, and holistic care. The opportunities presented by this innovative approach are promising and essential as we strive to align nutrition education and practice with the complex realities of our diverse and ever-changing world. The move toward ICM is a step toward a future in which food is understood and celebrated as a vital component of our physical, social, spiritual, and emotional well-being.

## Integrating ICM into Nutrition Curriculum: Challenges and Next Steps

The promising potential of using an ICM framework in nutrition pedagogy brings obstacles and opportunities. As health care stands on the cusp of re-envisioning health and care through the lens of nutrition education, it is worth exploring at an early phase how concepts from the ICM model might inform new curricula. As we navigate the path forward, there are important considerations, with the next steps being driven by the learner's needs.

One significant hurdle is adapting the ICM framework to the existing curriculum, aligning it with current nutrition education standards and practices, and finding time in an already busy schedule where nutrition may be undervalued. The next steps to overcome this obstacle include conducting a comprehensive review of existing curricula to identify areas where ICM principles can be integrated without overwhelming the existing schedule, and collaborating with educators and administrators to emphasize the value of nutrition education and create space for ICM within the curriculum.

The development of learning objectives and materials reflecting the multifaceted approach of ICM presents another difficulty. Utilizing the ICM framework as a reference to develop clear and comprehensive learning objectives and create engaging materials that encompass the integrative philosophy of ICM are essential next steps in this area.

Training and support for educators also pose a challenge, as equipping them with the knowledge and skills to effectively teach ICM principles is vital. Developing specialized training programs, providing ongoing support for educators, and fostering a community of practice to encourage collaboration and continuous learning are crucial next steps.

Finally, the evaluation and continuous improvement of the ICM-integrated curriculum is a complex task. Implementing regular evaluations, including student assessments and feedback from educators, and utilizing findings to refine and enhance the curriculum continually are crucial next steps.

## Closing

In conclusion, expanding nutrition education to include concepts of ICM not only offers exciting possibilities in fostering a salutogenic approach with food and diet but also requires careful consideration of various challenges. The path forward involves thoughtful planning, collaboration, and a commitment to elevating the value of nutrition education within the broader curriculum. ICM provides an opportunity to nurture a culture of wellness that recognizes food not just as a source of nutrition but also as a vital component of our physical, social, spiritual, and emotional well-being. The potential to transform nutrition education and promote a well-being culture is exciting and attainable.
